# The role of business size in assessing the uptake of health promoting workplace initiatives in Australia

**DOI:** 10.1186/s12889-016-3011-3

**Published:** 2016-04-21

**Authors:** A. W. Taylor, R. Pilkington, A. Montgomerie, H. Feist

**Affiliations:** Population Research & Outcome Studies, Discipline of Medicine, The University of Adelaide, Adelaide, South Australia; School of Public Health, The University of Adelaide, Adelaide, South Australia; Discipline of Medicine, The University of Adelaide, Adelaide, South Australia; Australian Population & Migration Research Centre, The University of Adelaide, Adelaide, South Australia

**Keywords:** Worksite health promotion, Australia, Business size

## Abstract

**Background:**

Worksite health promotion (WHP) initiatives are increasingly seen as having potential for large-scale health gains. While health insurance premiums are directly linked to workplaces in the USA, other countries with universal health coverage, have less incentive to implement WHP programs. Size of the business is an important consideration with small worksites less likely to implement WHP programs. The aim of this study was to identify key intervention points and to provide policy makers with evidence for targeted interventions.

**Methods:**

The worksites (*n* = 218) of randomly selected, working participants, aged between 30 and 65 years, in two South Australian cohort studies were surveyed to assess the practices, beliefs, and attitudes regarding WHP. A survey was sent electronically or by mail to management within each business.

**Results:**

Smaller businesses (<20 employees) had less current health promotion activies (mean 1.0) compared to medium size businesses (20–200 employees – mean 2.4) and large businesses (200+ employees – mean 2.9). Management in small businesses were less likely (31.0 %) to believe that health promotion belonged in the workplace (compared to 55.7 % of medium businesses and 73.9 % of large businesses) although half of small businesses did not know or were undecided (compared to 36.4 and 21.6 % of medium and large businesses). In total, 85.0 % of smaller businesses believed the health promotion activities currently employed in the worksite were effective (compared to 89.2 % of medium businesses and 83.1 % of large businesses). Time and funding were the most cited responses to the challenges to implementing health promoting strategies regardless of business size. Small businesses ranked morale and work/life balance the highest among a range of health promotion activities that were important for their workplace while work-related injury was the highest ranked consideration for large businesses.

**Conclusion:**

This study found that smaller workplaces had many barriers, beliefs and challenges regarding WHP. Often small businesses find health promotion activities a luxury and not a serious focus of their activities although this study found that once a health promoting strategy was employed, the perceived effectiveness of the activities were high for all business regardless of size. Tailored low-cost programs, tax incentives, re-orientation of work practices and management support are required so that the proportion of small businesses that have WHP initiatives is increased.

## Background

Extensive literature exists on the benefits, costs and barriers of worksite health promotion (WHP) initiatives. Research has highlighted many benefits of WHP including improved overall health, reduced absenteeism and pre-absenteeism, increased job satisfaction, more productive workers, and increased morale [1–5]. The benefits also include a decreased burden on the public health system [6]. As working participation rates are over 60 % for most western democratic countries [7–9], and with a large part of the day of the working population usually spent at a workplace, there consists a unique opportunity of implementing changes in health behaviours in workplace settings. The workplace, with already established social and organisation structures and proven methods of communication, is increasingly seen by many governments and health promotion agencies as ideal settings for large-scale health gains [9–12].

One of the major issues assessed is the uptake of WHP by size of workplace. Evidence suggests smaller firms are less likely to have successfully initiated health promotion interventions [10, 13–16] compared to the larger workplaces who have the benefits of economies of scale and additional resources [13]. The research indicates that implementation of WHP decreases as the size of the workplace decreases [17] with the most cited barriers for smaller workplaces being lack of interest from workers, lack of knowledge, program costs, lack of appropriate level of resources and lack of support from management [10, 14, 16]. In addition, privacy concerns are more likely to be an issue in smaller workplaces [16, 18]. While the most popular WHP initiatives include assistance with smoking cessation, increasing physical activity, improving nutrition, stress management and reduction, weight management, and responsible alcohol intake, the uptake also vary by size of workplace.

In the United States (USA) health insurance premiums are directly linked to workplaces and paid for by employers, but the system and experience is different for many other countries. Australia, Canada and the United Kingdom have variations of universal health care coverage. As such, WHP programs in Australian workplaces, for example, are somewhat limited as companies often fail to see the links between work and health outcomes; with less financial interest in implementing any changes or programs [2, 4]. Since the WHO Health Workforce Decade (2006–2015) was implemented, some changes are apparent in Australia with increased government incentives for companies to embrace WHP. Worldwide there is increased literature gradually citing return on investments, regardless of health insurance payment criteria [10].

Senior management support has been a contributory factor to successful WHP programs and is linked to business size [9, 10, 19–21] although other research has indicated that WHP success relies on being driven by workers rather than management *per se* [18]. Notwithstanding, the involvement and participation of employees and management working together with shared visons and agreed initiatives are more likely to succeed although the level of involvement often differs by business size [22]. While research has shown that over three quarters of managers believe health promotion programs are very important, other important differences such as perceived benefits and barriers were apparent [23]. Most of these cited publications are from USA, where cost is an important attribute, associated with overall increased health spending, and subsequently an interest at all levels of management in reducing costs and hence increasing preventive opportunities [13]. Differences in commitment and perceptions of the value of WHP still exist.

Evidence suggest that uptake of long-term workplace directed practices are often limited [13] and additional robust research is required [9, 24]. Research on the influence of business size and management involvement has also been called for, especially in Australia [3, 16, 24]. This study has surveyed workplaces of participants in two long-standing cohort studies to characterise the workplaces by size of business, so that the uptake, commitment, attitude and beliefs regarding policy and practice of WHP initiatives can be assessed in an Australian setting. The overall aim of the project was to identify key intervention points and to provide policy makers with evidence for targeted interventions.

## Methods

Australia’s Baby Boomer Generation, Obesity and Work – Patterns, Causes and Implications was an Australian Research Council funded project. As a part of this project, in October 2011 a telephone survey was undertaken with eligible participants from two cohort studies - the North West Adelaide Health Study (NWAHS) and the Florey Adelaide Male Ageing Study (FAMAS). The content of the survey was based on WHP literature and previous surveys addressing barriers and enablers to the implementation of WHP initiatives. Detailed descriptions of the methodology and cohorts ares available elsewhere [25–27].

In the 2011 telephone survey, eligible respondents from the two methodologically similar cohorts who were born between 1946 and 1980 (aged 30 to 65 years), had worked in the previous three years, were not self-employed, were currently working or knew their employer’s details were asked to name their place of work with 69.1 % (*n* = 956) supplying details. A questionnaire, a letter, and reminder letter (if required), addressed to the Managing Director, Chief Executive Officer or Human Resources representative of the organisation, was sent inviting them to participate in the workplace survey. A paper survey with a reply paid envelope was included in the package and respondents also had the option to complete the survey online with an allocated identification number. The letter outlined the importance of study, invited them to participate and ensured confidentiality. All business types (public/private) from all sectors were eligible for inclusion. Data were obtained from a total of *n* = 218 businesses representing *n* = 330 individuals (several business had more than one participant) (response rate = 34.5 %). Business size (small 2–19 employees; medium 20–200 employees; and large 200+ employees) as defined by Australian Bureau of Statistics classifications [28] was determined for this analysis from the responses in this questionnaire.

Data were collected on number of employees, whether occupational health and safety (OHS) or staff wellbeing were regular meeting agenda items (yes/no), who had responsibility for staff health and wellbeing (owner, managing director/CEO, OHS manager, human resources manager, the staff themselves, no one), and the number and type of health promotion activities currently undertaken (smoking cessation, alcohol and substance abuse, healthy food policy, physical activity, mental health and wellbeing, none, other). The employer’s representative was also asked if they believed the health promotion activities undertaken by the company were effective (very, somewhat, not, undecided), whether they believed that specific workplace health promotion programs belonged in the workplace (yes, no, undecided, don’t know) and what challenges they faced implementing these activities (funding, time, facilities, lack of management/board support, not a workplace responsibility). Respondents were also asked the perceived effect on the business if five specified health promotion programs (physical activity, healthy eating, alcohol, mental health, smoking) were introduced (already have program, adverse effect, no effect, positive effect, don’t know - with positive responses reported); to rank the extent to which they thought a range of 10 issues including work related injury, stress or burnout, alcohol, morale and work/life balance affected their workplace (with 1 = not very important and 5 = very important), and what resources they would require to increase physical activity and/or healthy eating in the workplace (written guidelines, government funding, best practice standards, consultants, information or advice about providers in the local area).

The survey was piloted tested (*n* = 10) and changes made based on the findings. The conventional 5 % level was used to determine statistical significance and 95 % confidence intervals were provided for estimates. Uni-variable analyses using chi-square (*χ*2) tests were undertaken to analyses differences in WHP practice and belief between businesses in terms of business size. Ethics approval for the workplace survey was gained by The University of Adelaide Human Research Ethics Committee.

## Results

In total, 19.3 % of responses (*n* = 42) were from small business (2–19 employees), 40.3 % (*n* = 88) from medium businesses (20–200 employees) and 40.3 % (*n* = 88) from large employers (over 200 employees).

The proportion of businesses that had regular OHS meetings, included staff wellbeing as an agenda item on management meetings and the number of health promoting activities undertaken in each of the businesses are listed in Table 1. Small businesses were less likely to have OHS agenda items and large businesses were less likely to have staff wellbeing discussed at management meetings. Small businesses were more likely to have no health promotion avtivities. The mean number of health promotion activities undertaken by business size was 1 (median 0; sd 1.255) for small businesses, 2.4 (medium 2; sd 1.491) for medium businesses and 2.9 (medium 3; sd 1.476) for large businesses. Table 1 also highlights the proportion believing that health promotion programs belong in the workplace. Overall more small businesses (19.0 %) believed that health-promoting activities did not belong in the workplace (with a further 50 % undecided or not knowing).

**Table 1:** Characteristics of businesses by business size

	Overall		Small		Medium		Large	
	n	% (95 % CI)	n	% (95 % CI)	n	% (95 % CI)	n	% (95 % CI)
Occupational health and safety as a regular management meeting agenda item	193	88.5 (83.6–92.1)	27	65.9 (50.5–78.4)^a^	85	97.7^b^	81	92.0 (84.5–96.1)
Staff wellbeing as a regular management meeting agenda item	118	54.1 (47.5–60.6)	21	51.2 (36.5–65.7)	56	64.4 (53.9–73.6)^a^	41	46.6 (36.5–56.9)
Number of current health promoting strategies								
None/not stated	45	20.6 (15.8–26.5)	21	51.2 (36.5–65.7)^a^	11	12.6 (7.2–21.2)^a^	11	12.5 (7.1–21.0)^a^
1–2 current health promoting strategy	84	38.5 (32.3–45.1)	14	34.1 (21.6–49.5)	39	44.8 (34.8–55.3)	31	35.2 (26.1–45.6)
3 or more current health promoting strategies	89	40.8 (34.5–47.5)	6	14.6 (6.9–28.4)^a^	37	42.5 (32.7–53.0)	46	52.3 (42.0–62.4)^a^
Do specific health promotion programs belong in the workplace?								
Yes, they belong in the workplace	127	58.3 (51.6–64.6)	13	31.0 (19.1–46.0)^a^	49	55.7 (45.3–65.6)	65	73.9 (63.8–81.9)^a^
No, they do not belong in workplace	19	8.7 (5.7–13.2)	8	19.0 (10.0–33.3)^a^	7	8.0 (3.9–15.5)	4	4.5^b^
Don’t know/Undecided/Not applicable/Not stated	72	33.0 (27.1–39.5)	21	50.0 (35.5–64.5)^a^	32	36.4 (27.1–46.8)	19	21.6 (14.3–31.3)^a^
Overall	218	100.0	42	100.0	88	100.0	88	100.0

^a^Statistically significantly different to other categories combined - *χ*2 test, *p* < 0.05

^b^Insufficient numbers for statistical test

Table 2 lists the health promotion activities undertaken. Small businesses were less likely to undertake any of the nominated activities. Large businesses were more likely to have most of the WHP strategies; healthy food, and mental health and wellbeing policies were more likely in medium size businesses. ‘Other’ health promotion activities included lifestyle payment incentives, health checks, employee assistance programs and lunch time presentations. Table 2 also highlights who respondents perceived should have responsibilities for staff health and welfare with over 90 % of large businesses stating ‘staff themselves’ and 85.4 % stating management. Multiple responses were allowed. In all instances except for the ‘business owner’ category, the positive response decreased as size of business decreased. The barriers to implementing changes in the workplace are also detailed in Table 2 with ‘time’ the most prevalent response for each business size. ‘Funding’ was the second most challenging aspect for all businesses.

**Table 2:** Type of health promotion activities, responsibility for OHS and challenges to implementation, by business size

	Overall		Small		Medium		Large	
	n	% (95 % CI)	n	% (95 % CI)	n	% (95 % CI)	n	% (95 % CI)
Current health promotion activities^a^								
Smoking cessation policy	106	51.0 (44.2–57.7)	11	26.8 (15.7–41.9)^b^	47	54.7 (44.2–64.7)	48	59.3 (48.4–69.3)
Alcohol and substance misuse	116	55.8 (49.0–62.4)	13	31.7 (19.6–47.0)^b^	44	51.2 (40.8–61.4)	59	72.8 (62.3–81.3)^b^
Healthy food policy	61	29.3 (23.6–35.8)	5	12.2 (5.3–25.5)^b^	32	37.2 (27.7–47.8)^b^	24	29.6 (20.8–40.3)
Physical activity	64	30.8 (24.9–37.3)	2	4.9^bc^	27	31.4 (22.6–41.8)	35	43.2 (33.0–54.1)^b^
Mental health and wellbeing	105	50.5 (43.7–57.2)	11	26.8 (15.7–41.9)^b^	50	58.1 (47.6–68.0)	44	54.3 (43.5–64.7)
Other health promotion activities	34	16.3 (11.9–22.0)	–	–	8	9.3 (4.8–17.3)^b^	26	32.1 (22.9–42.9)^b^
None/not stated	45	20.6 (15.8–26.5)	21	51.2 (36.5–65.7)^b^	11	12.6 (7.2–21.2)^b^	11	12.5 (7.1–21.0)^b^
Who has responsibility for staff health and wellbeing^a^								
Business owner	66	31.6 (25.7–38.2)	15	36.6 (23.6–51.9)	29	33.3 (24.3–43.8)	22	27.2 (18.7–37.7)
Managing director/CEO	153	72.9 (66.5–78.4)	22	53.7 (38.7–67.9)^b^	61	70.1 (59.8–78.7)	70	85.4 (76.1–91.4)^b^
Occupational Health and Safety manager	123	58.6 (51.8–65.0)	12	29.3 (17.6–44.5)^b^	51	58.6 (48.1–68.4)	60	73.2 (62.7–81.6)^b^
Human Resource Manager	104	49.5 (42.8–56.2)	10	24.4 (13.8–39.3)^b^	33	37.9 (28.5–48.4)^b^	61	74.4 (64.0–82.6)^b^
The staff themselves	172	81.9 (76.1–86.5)	28	68.3 (53.0–80.4)^b^	70	80.5 (70.9–87.4)	74	90.2 (81.9–95.0)^b^
No one in particular	1	0.5^c^	1	2.4^c^	–	–	–	–
Challenges to implementing health promoting strategies by business size^a^								
Funding	129	62.0 (55.3–68.3)	17	42.5 (28.5–57.8)^b^	55	64.0 (53.4–73.3)	57	69.5 (58.9–78.4)
Time	162	77.9 (71.8–83.0)	31	77.5 (62.5–87.7)	68	78.2 (68.4–85.5)	63	77.8 (67.6–85.5)
Facilities	71	34.3 (28.2–41.0)	13	32.5 (20.1–48.0)	31	36.0 (26.7–46.6)	27	33.3 (24.0–44.1)
Lack of management/board support	25	12.1 (8.4–17.3)	9	22.5 (12.3–37.5)	8	9.3 (4.8–17.3)	8	10.0 (5.2–18.5)
Not a workplace responsible	12	5.8 (3.4–9.9)	5	12.5 (5.5–26.1)	4	4.7^c^	3	3.8^c^

*CI* confidence interval

^a^Multiple response

^b^Statistically significantly different to other categories combined - *χ*2 test, *p* < 0.05

^c^Insufficient numbers for a statistical test

Table 3 lists the proportion of businesses believing a positive effect would result if specific health promoting programs were introduced with small businesses less likely to report a positive effect for each of the five nominated programs. Large businesses had the most favourable responses for each activity except for physical activity where 60 % of medium size businesses believed it had a positive effect on their business.

**Table 3:** Belief that Health Promotion programs have a positive effect on business, by business size

	Overall		Small		Medium		Large	
	n	% (95 % CI)	n	% (95 % CI)	n	% (95 % CI)	n	% (95 % CI)
Physical activity	110	51.9 (45.2–58.5)	19	45.2 (31.2–60.1)	51	60.0 (49.4–69.8)	40	47.1 (36.8–57.6)
Healthy Eating	109	51.4 (44.7–58.1)	18	42.9 (29.1–57.8)	46	52.9 (42.5–63.0)	45	54.2 (43.5–64.5)
Alcohol programs	83	39.3 (33.0–46.1)	12	28.6 (17.2–43.6)	28	32.9 (23.9–43.5)	43	51.2 (40.7–61.6)^a^
Mental Health programs	112	53.3 (46.6–60.0)	17	40.5 (27.0–55.5)	44	52.4 (41.8–62.7)	51	60.7 (50.0–70.5)
Smoking policy	85	40.3 (33.9–47.0)	14	33.3 (21.0–48.4)	34	40.5 (30.6–51.2)	37	43.5 (33.5–54.1)

Unweighted data. *CI* confidence interval. ^a^Statistically significantly different to other categories combined - *χ*2 test, *p* < 0.05

Table 4 details the results for proportion of the businesses that reported currently undertaking health promotion activities (*n* = 171). Of those, 85.0 % of small business believed they were somewhat or very effective (compared to 89.2 % of medium businesses and 83.1 % of large businesses).

**Table 4:** Perceived effectiveness of health promotion activities by business size

	Overall		Small		Medium		Large	
	n	% (95 % CI)	n	% (95 % CI)	n	% (95 % CI)	n	% (95 % CI)
Very effective	16	9.4 (5.8–14.7)	3	15.0^a^	8	10.8 (5.6–19.9)	5	6.5 (2.8–14.3)
Somewhat effective	131	76.6 (69.7–82.3)	14	70.0 (48.1–85.5)	58	78.4 (67.7–86.2)	59	76.6 (66.0–84.7)
Not effective/Undecided	24	14.0 (9.6–20.0)	3	15.0^a^	8	10.8 (5.6–19.9)	13	16.9 (10.1–26.8)
Overall	171	100.0	20	100.0	74	100.0	77	100.0

^a^Insufficient numbers for a statistical test. Unweighted data. CI: Confidence Interval

The mean response to the question regarding whether a range of issues was important for their workplace is detailed in Table 5. The mean response increased as business size increased for work related injury, smoking, alcohol and overweight/obesity and conversely fell by increased business size for morale. Morale received the highest mean score of the 10 issues measured for medium businesses (mean 3.9 sd = 1.06) while work related injury (mean 4.0 sd 1.21) was the highest ranked issue for large businesses and work life balance (mean 4.2 sd 0.99) for small businesses.

**Table 5:** Belief that health promotion activities are important for the workforce, by business size (1 = not important; 5 = very important)

	n	Work related injury	Lack of sleep/alertness	Stress or burnout	Smoking	Alcohol	Obese/Overweight	Chronic/ageing related problems	Mental health conditions	Morale	Work/life balance
		Mean ± SD	Mean ± SD	Mean ± SD	Mean ± SD	Mean ± SD	Mean ± SD	Mean ± SD	Mean ± SD	Mean ± SD	Mean ± SD
Overall	215	3.8 ± 1.365	3.5 ± 1.122	3.8 ± 1.059	2.6 ± 1.196	2.8 ± 1.230	3.1 ± 1.076	3.0 ± 1.157	3.3 ± 1.116	3.8 ± 1.068	3.9 ± 0.954
Small	41	3.6 ± 1.566	3.6 ± 1.163	3.8 ± 1.116	2.3 ± 1.209	2.6 ± 1.337	2.9 ± 1.038	3.0 ± 1.301	3.1 ± 1.321	4.0 ± 1.203	4.2 ± 0.997
Medium	87	3.8 ± 1.407	3.4 ± 1.177	3.8 ± 1.091	2.5 ± 1.247	2.8 ± 1.271	3.1 ± 1.153	2.9 ± 1.178	3.1 ± 1.023	3.9 ± 1.063	3.8 ± 0.938
Large	87	4.0 ± 1.210	3.4 ± 1.050	3.9 ± 1.010	2.7 ± 1.120	2.9 ± 1.140	3.3 ± 1.000	3.2 ± 1.070	3.5 ± 1.090	3.8 ± 1.010	3.8 ± 0.940

*SD* standard deviation. Unweighted data

Table 6 highlights the various resources businesses stated they would require to help them introduce various WHP initiatives. The requirements did not differ markedly by business size.

**Table 6:** Resources required to introduce a health promotion program focusing on increasing physical activity, or healthy eating, or both, by business size^a^

	Overall		Small		Medium		Large	
	n	% (95 % CI)	n	% (95 % CI)	n	% (95 % CI)	n	% (95 % CI)
Written guidelines	113	52.8 (46.1–59.4)	21	51.2 (36.5–65.7)	41	47.1 (37.0–57.5)	51	59.3 (48.7–69.1)
Government grants/funding	116	54.2 (47.5–60.7)	22	53.7 (38.7–67.9)	46	52.9 (42.5–63.0)	48	55.8 (45.3–65.8)
Best practice standards	107	50.7 (44.0–57.4)	17	41.5 (27.8–56.6)	41	47.7 (37.4–58.1)	49	58.3 (47.7–68.3)
Consultants	86	40.6 (34.2–47.3)	16	39.0 (25.7–54.3)	34	39.5 (29.9–50.1)	36	42.4 (32.4–53.0)
Information or advice about providers in the local area	113	53.3 (46.6–59.9)	19	46.3 (32.1–61.3)	47	54.0 (43.6–64.1)	47	56.0 (45.3–66.1)

Unweighted data. *CI* confidence interval. ^a^Multiple response

## Discussion

The businesses assessed in this study were the workplaces of participants who were randomly selected to be included in two major cohort studies undertaken in South Australia. These businesses were classified into business size based on number of employees and results indicate that smaller workplaces have many barriers, beliefs and challenges to having health promoting workplaces.

In line with many other major studies, the overall implementation of evidence-based WHP initiates in small businesses was low [17, 29]. Over 50 % of small businesses have not embraced WHP concepts and lag behind markedly in the health-promoting activities that we specifically assessed. In common with USA studies where estimates of over 90 % of workplaces offer at least one health promoting activity [4], our study found that over 86 % of medium or large workplaces embraced at least one health promotion strategy. One of the common benefits of using a work place as a health-promoting site is based on the existing ‘community-like’ environment often found at worksites [8]. While this may be true for larger businesses, most small businesses find these policies/activities a luxury, not central to their main focus [10] and lack the infrastructure, incentives and perceived benefits to such activities. Changing these notions is a first step with many small business operators lacking the knowledge and experience to make the first step in the required changes [10].

Common barriers such as cost, time and travel associated with many other forms of community-wide health promotion interventions, are typically reduced with WHP campaigns. This is not necessarily the case with small businesses, as shown in this and other studies [16], where cost is a major reason for the lack of health promoting initiatives. The financial risk often associated with many aspects of operating a small business means that implementing perceived costly health promotion interventions receive a low priority. Such concepts as including use of company facilities, equipment and other infrastructure, and having environmentally suitable workplaces that encourages healthy activities (stairwells, healthy food supply) [14] are not practical, feasible or cost efficient within a small workplace. However as argued by Linnan [15], the growing evidence that WHP programs lead to broad-reaching improvements such as better overall employee health, morale and productivity may provide additional benefits not normally considered by small business operators and should be encouraged and promoted. Small businesses also have fewer personnel that can be assigned the responsibility of promoting these activities or guidelines or even highlighting them as an OHS consideration [15]. Notwithstanding, 85 % of our sample of small business operators/managers who had health promoting activities in their workplace, believed that their current promotion activities were effective, and believe in the value of the programs, highlighting that once adopted, positive sentiments prevail. Those small businesses who take up health promotion activities are able to see or measure the benefits. Combine this with the perceived need for resources being similar to that for all businesses suggests that we could use these ‘small business champions’ to spread the word about the positive impacts for small businesses and the creative ways they have overcome resource needs to make this happen.

The importance of the business owner in implementing change is highlighted with management support being the main challenge to implementing WHP strategies. Notably, our figure of 74 % of large businesses believing WHP to be important corresponds with other literature [23]. While other studies have shown high levels of managers believing health promotion programs to be important, [21, 23], only 31 % of small businesses in this study believed they belong in the workplace. While small business operators are not convinced health promotion programs in the workplace are effective with one in five not believing health promoting activities belong in the workplace, a further 50 % reported that they ‘did not know’ or were ‘undecided’. This latter group are a potential target to influence, especially their beliefs and knowledge about the benefits of WHP. In addition, small business respondents to this survey were less likely to report positive effects of specific health promoting programs than larger businesses with the most positive response being for physical activity (45.2 %) and healthy eating (42.9 %). In contrast, only 28.6 % of small businesses could see a positive effect of introducing an alcohol program.

An important finding of this study was the relative importance of work-life balance issues with nearly 50 % of small business respondents ranking work/life balance as a very important issue. Implementing WHP initiatives that encourage work-life balance as well as addressing the broader health and wellbeing agenda in small businesses is warranted [2]. Highlighting the benefits of health promotion programs that assist in self-management to increase knowledge and coping skills, and make ‘the healthy choices the easy choices’, is also warranted [1]. Morale, work life balance and stress all ranked highly in terms of importance for health promotion. These are clearly linked more closely to work productivity, or have received more attention than other issues. One questions whether issues such as smoking and alcohol are a low priority because they are not seen as influencing worker’s health and productivity as they are not allowed in work places, or whether people think there is already enough health promotion in this area.

Weaknesses of the study included the use of self-reported measures rather than audit techniques with potential bias and self-affirmation included in the response especially as there was no explicit instructions on whom within the company should complete the questionnaire. A further weakness was the limiting of the health promoting activities to six activities while excluding activities such as influenza vaccinations and stress management. There was also no economic assessment undertaken and no assessment of concern with privacy or breaches of confidentiality often cited as a major barrier to investing in WHP principles, especially in small businesses, and highlighted extensively in the literature [10, 18]. The definition of what constitutes a small business differs across countries and studies [23] making comparison difficult and the use of Australian Bureau of Statistics classifications of business size as arbitrary cut-offs may have limited other associations. In addition, there was a potential bias from survey non-response in this study with a response rate of 35 % and this should be seen as a weakness of the study. An additional weakness was the lack of power to link individual health outcomes to WHP workplace initiatives. No analysis was undertaken to assess the representativeness of the sample and as such the results could be biased although a relatively large number of businesses and business types were included. As the initial sample was from a cohort of people aged 30+ years and included more females than males bias may of resulted and generalizability of the results may have been compromised. In addition, validity and reliability of the questions used in the survey were un-determined. The strengths of this study include the random nature of the selection of worksites without being limited to pre-determined industry or sector-wide business types, the wide range of questions asked and the relatively large number of business assessed. The ability to eventually link the participants’ health outcomes over the life of the cohort study to workplace characteristics has much potential.

This research has shown that while workplaces on the whole are embracing health-promoting activities in the workplaces, small business and hence workers employed by small business operators, are lagging behind in implementing the required changes. Management support and change of beliefs are required for ultimate and sustained success [20]. Success will not be obtained without reorienting work practices and in small businesses this reorientation is not necessarily a priority. Commitment to changes in policies, systems and providing resources may be required [30]. Heinen [14] and others [10, 15] call on government policy interventions and financial incentives (including tax incentives) for small-based workplaces in an endeavour to increase WHP initiatives and to accelerate change. These may well be warranted. Given that funding was listed as a challenge to WHP implementation by over 60 % of businesses, tax incentives may have a significant impact on WHP. Others have argued that facilitating a culture within a workplace is often a cost neutral endeavour that can ultimately have positive effects [14]. As argued by Linnan et al. [15], and supported by these analysis, different levels of workplaces perhaps require different types of programs, policies and practices as a ‘one-size fits all approach’ is unlikely to be successful [31].

## Conclusions

Within Australia WHP is an ongoing process with recent encouragement from government initiatives [31]. This study has filled a void on research on the requirements and needs of small businesses. The need for low cost, low-resourced, creative activities and solutions and more detailed knowledge of the benefits to employees are required [18, 31]. Corresponding knowledge improvement via mass media campaigns and promotions will also be beneficial. While the results of this research have highlighted the low uptake of health promotion initiatives in small workplaces, some positive results are evident. These include the proportion of small businesses that have WHP, responding favourably to their influence and effectiveness and the positive results for larger businesses both of which herald encouraging signs for the health of the workers of Australia.

## Ethics and consent to participate

Ethical approvals were obtained from the Research Ethics Committee of The University of Adelaide. Participation in the study was voluntary. Verbal informed consent was obtained from participants at the start of the interview. Prior to contact by the interviewers, a primary approach letter was sent informing them of the purpose of the survey including details on confidentiality and privacy, that participation was voluntary, and a contact number for queries. Participants were able to refuse to answer any questions or terminate the interview at any point.

## Consent to publish

Not applicable.

## Availability of data and materials

Data are available, on application, from the corresponding author.

## Abbreviations

FAMAS: Florey Adelaide Male Ageing Study
NWAHS: North West Adelaide Health Study (NWAHS)
OHS: occupational health and safety
WHO: World Health Organization
WHP: worksite health promotion
